# Discovery and mapping of single feature polymorphisms in wheat using Affymetrix arrays

**DOI:** 10.1186/1471-2164-10-251

**Published:** 2009-05-29

**Authors:** Amy N Bernardo, Peter J Bradbury, Hongxiang Ma, Shengwa Hu, Robert L Bowden, Edward S Buckler, Guihua Bai

**Affiliations:** 1 Dept. of Plant Pathology, Kansas State University, Manhattan, KS 66506, USA; 2 ARS-USDA, Maize Genetic Diversity Laboratory, Ithaca, NY 14853, USA; 3 Institute of Plant Genetics and Biotechnology, JAAS, Nanjing, PR China; 4 Dept. of Agronomy, Kansas State University, Manhattan, KS 66506, USA; 5 ARS-USDA Plant Science and Entomology Unit, Manhattan, KS 66506, USA

## Abstract

**Background:**

Wheat (*Triticum aestivum *L.) is a staple food crop worldwide. The wheat genome has not yet been sequenced due to its huge genome size (~17,000 Mb) and high levels of repetitive sequences; the whole genome sequence may not be expected in the near future. Available linkage maps have low marker density due to limitation in available markers; therefore new technologies that detect genome-wide polymorphisms are still needed to discover a large number of new markers for construction of high-resolution maps. A high-resolution map is a critical tool for gene isolation, molecular breeding and genomic research. Single feature polymorphism (SFP) is a new microarray-based type of marker that is detected by hybridization of DNA or cRNA to oligonucleotide probes. This study was conducted to explore the feasibility of using the Affymetrix GeneChip to discover and map SFPs in the large hexaploid wheat genome.

**Results:**

Six wheat varieties of diverse origins (Ning 7840, Clark, Jagger, Encruzilhada, Chinese Spring, and Opata 85) were analyzed for significant probe by variety interactions and 396 probe sets with SFPs were identified. A subset of 164 unigenes was sequenced and 54% showed polymorphism within probes. Microarray analysis of 71 recombinant inbred lines from the cross Ning 7840/Clark identified 955 SFPs and 877 of them were mapped together with 269 simple sequence repeat markers. The SFPs were randomly distributed within a chromosome but were unevenly distributed among different genomes. The B genome had the most SFPs, and the D genome had the least. Map positions of a selected set of SFPs were validated by mapping single nucleotide polymorphism using SNaPshot and comparing with expressed sequence tags mapping data.

**Conclusion:**

The Affymetrix array is a cost-effective platform for SFP discovery and SFP mapping in wheat. The new high-density map constructed in this study will be a useful tool for genetic and genomic research in wheat.

## Background

In many plant species, high-resolution mapping of genes is limited by lack of sufficient DNA markers. This limitation is especially significant when quantitative trait loci (QTLs) control a trait because QTLs may remain undetected or their effects may be underestimated when marker density is low. Linkage disequilibrium (LD) maps and association mapping also require the identification of many markers at very high resolution from many different individuals. Marker-assisted breeding is another application that requires abundant markers for integration of genes/traits into modern crop varieties.

Single nucleotide polymorphisms (SNPs) are abundant and provide a rich source of potential DNA markers. Individual SNPs may also directly contribute to phenotypic variation if they are in an intragenic or promoter region [1,2] and can be used as perfect markers for genes/traits of interests. In addition to their abundance, SNPs have the advantage of several high throughput genotyping platforms that significantly reduce the cost per data point. In soybean, resequencing sequence-tagged sites derived from ESTs led to discovery of SNPs, and a map consisting of 1,141 SNP loci was generated using three RIL populations [3]. Similarly, a barley map made of 300 SNP loci was constructed using SNPs developed from resequencing unigenes [4].

Although bread wheat (*Triticum aestivum *L.) is also a major world food crop, progress on SNP discovery has been slow compared to soybean and model organisms such as Arabidopsis and rice [5,6]. The wheat genome has not yet been sequenced due to the huge genome size (~17,000 Mb) and because it contains about 80% repetitive sequences [7]. Wheat is an allohexaploid with 21 chromosomes consisting of seven homoeologous chromosomes from each of three ancestral genomes (A, B, D). The three genomes are closely related, which complicates SNP analysis of homoeologous gene sequences [8]. Wheat generally has low sequence polymorphism as a consequence of bottlenecks encountered during polyploidization and domestication [9]. Large expressed sequence tag (EST) databases have been developed for wheat and these have been successfully mined for SNPs using contig alignments and/or resequencing [8,10,11]. However, the number of SNPs available for genotyping in wheat is still relatively small and many SNPs are only polymorphic in wild wheat relatives [11]. New technologies that detect genome-wide polymorphisms in wheat are needed to discover a large number of new markers for genomic research and breeding in wheat.

SNPs and insertion or deletions of one or more nucleotides (indels) are DNA polymorphisms that can affect hybridization of DNA or cRNA to a probe on an array. The Affymetrix GeneChip arrays are suitable to detect such variations because each gene is represented by a set of eleven 25-bp probes that are sensitive to target mismatch owing to their short sequence. A target sequence that perfectly matches the sequence of a probe binds with much greater affinity than one with a mismatch sequence. The resulting difference in hybridization intensity between two genotypes for an individual probe is called a single feature polymorphism (SFP), where a feature refers to a probe in the array. A SFP may be caused by a SNP, a multiple nucleotide polymorphism, or an indel. However, if cRNA is used for hybridization, gene expression markers (GEMs) that reflect expression level differences may also be detected [12,13].

Winzeler et al. first described the method for detection of SFPs by hybridizing DNA from different yeast strains to high-density oligonucleotide arrays [14]. They identified 3,714 markers that were used for high-resolution mapping of five loci in yeast. Using the same approach, about 4,000 SFPs were identified between two *A. thaliana *strains by using the AtGenome1 GeneChip [15]. DNA sequence alignment of AtGenome1 feature sequences with publicly available Arabidopsis sequence data confirmed that 117 out of 121 AtGenome1-predicted SFP have sequence variants. In addition, a known mutation was mapped by bulked segregant analysis, hybridization of pools of mutants and wild-types to the microarray [15]. Singer et al. used an array-based hybridization method to construct an SFP map in Arabidopsis containing 676 markers [16]. In barley, more than 10,000 SFPs were discovered using the Affymetrix Barley1 GeneChip [17]). Out of 450 barley SFPs, 270 were verified to contain SNPs by sequence comparison with barley sequence datasets [17]. A study by Kumar et al. detected 5,376 SFPs in rice between two *japonica *subspecies and 25,325 SFPs between *japonica *and *indica *subspecies [18].

Microarray hybridizations with genomic DNA may not be satisfactory for SFP discovery in species with large genomes [19]. Several studies successfully used labeled cRNA instead of genomic DNA to hybridize to the array to reduced background and enrich for expressed gene sequences [12,19,20]. In wheat, 297 SFPs were identified between near-isogenic lines contrasting in stripe rust resistance using the Affymetrix GeneChip Wheat Genome Array [21]. Gore et al. compared different target preparation methods to reduce target complexity [22]. They tested the Affymetrix Maize Genome Array for SFP detection using a set of 13,000 probes with known sequence. Results showed that the best enrichment method using all the Maize Genome Array data should be able to detect about 10,000 SNP in maize at a 20% false discovery rate. One shortcoming of this approach is that transcripts represented in cRNA pools can vary greatly between tissues, developmental stages, and treatments.

This research was designed to explore the utility of the Affymetrix GeneChip Wheat Genome Array for discovering and mapping SFPs in the large and complex hexaploid wheat genome. Pooled RNAs from two tissues were used to increase the diversity of transcripts and cRNA instead of DNA was used to minimize the problems of large genome size and repetitive DNA. A greater concern was the potential for interference between homoeologous or paralogous gene copies in probe hybridizations. Mochida et al. estimated that less than half of homoeologous genes were expressed by only one genome and many were expressed by all three genomes [8]. Akhunov et al. reported that one quarter of all wheat gene motifs were present in two or more paralogous copies [23]. The current study used two different strategies for reducing the problem of interference. In the first experiment, a panel of six diverse varieties of wheat was analyzed for probe × variety interactions. Cluster analysis was used to filter results and only biallelic SFPs with intermediate frequencies were retained. In the second experiment, SFPs in 71 recombinant inbred lines (RILs) from the cross of Ning 7840/Clark were analyzed. SFPs that gave clear allele calls in at least 60 RILs were selected for map construction.

## Results

### SFP among Six Wheat Varieties

**Figure 1:**
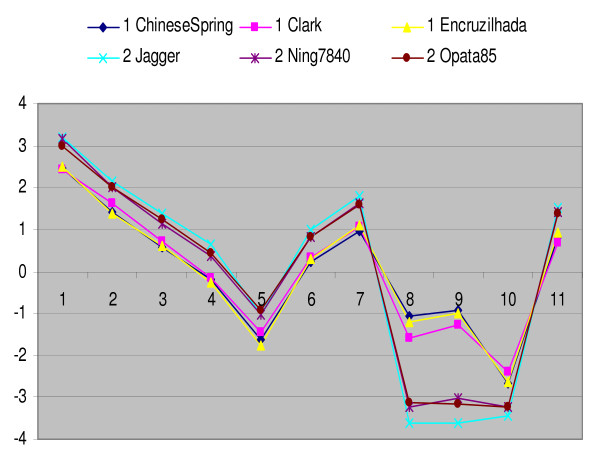
**Signal intensity of the 11 probes within the Ta.12752.2.S1_x_at probe set**. SFPs are detected by the difference in signal intensity between perfect match probes within a probe set for two genotypes. The difference between the 11 perfect match probes should remain constant between two genotypes if there is no polymorphism. When there is a SNP, hybridization between target and a particular probe is affected, and the difference in probe signal intensity between two genotypes changes. Probes 8, 9 and 10 are predicted to have sequence polymorphisms.

Out of the 61,127 probe sets analyzed on six wheat varieties, 19,896 had at least three probes with a signal intensity of 200 or more, and 3,769 of those probe sets (20%) had a *p*-value of < 1e^-10 ^for probe × variety interaction (Figure 1). K-means cluster analysis was done using SAS PROC FASTCLUS [24] on each of the 3,769 probe sets with the number of clusters set to 2. There were 396 probe sets with overall R-square (Rsq) ratio > 4 and minimum count > 1, where Rsq = proportion of the variability explained by the clusters, Rsq_ratio equals to Rsq/(1-Rsq), and minimum count is the number of varieties in the smallest cluster. This algorithm does not distinguish multiple SFPs in the same probe set. From this point forward, the term SFP refers to a probe set with at least one polymorphic probe.

### Sequence Variation of Selected SFPs

For designing primers, the Rsq_ratios for the individual probes within a probe set were considered and focus was on probes with the highest values of Rsq_ratios because those were driving the cluster formation most strongly and, therefore, were most likely to be polymorphic between varieties (Figure 1). Out of the 396 probe sets or genes selected, we were able to design primers for 359 genes. To investigate whether the SFPs were related to sequence variations, variety pairs were selected for sequencing based on clustering results. One hundred sixty-four primers (46%) amplified single bands and gave high quality DNA sequence in the variety pairs tested (Table 1). Most amplicons were 300–550 bp, but some were as short as 200 bp or as long as 1,800 bp. DNA sequence data was examined manually to identify polymorphisms that overlapped with the 25-mer probes. Sequencing data confirmed that 88 unigenes (Table 1) had a total of 139 sequence polymorphisms within the probes. The accuracy of the array to predict the presence of a sequence variant based on a SFP is 54% (88/164). An additional 353 sequence variants were discovered outside of the probe sequences, bringing the total count to 492 sequence polymorphisms representing 106 unigenes. In the 88 unigenes with SFPs, 75% of the sequence variations were SNPs, 10% were multiple nucleotide polymorphisms (adjacent SNPs), and 15% were indels. The indels ranged from 1 to 31 bp insertions or deletions. On average, every 112-bp of sequenced partial unigenes had a SNP, every 733-bp had a multiple nucleotide polymorphism and every 1048-bp had an indel.

The percentage of SFPs that were monomorphic with respect to sequence corresponding to the probe set is 46% (76/164). Out of these 76 unigenes, 18 had SNPs outside of the probe sequence, whereas 58 had identical sequence between the two varieties being compared. To further confirm the absence of sequence polymorphism, six out of the 58 genes were randomly selected and sequenced from all six varieties, and visual comparison of aligned sequences verified that all varieties tested shared identical sequences.

**Table 1:** Results of PCR and sequence analysis of SFPs in six wheat genotypes.

	Ning 7840 and Clark Variety Pair	Other Variety Pairs	Total
No. of unigenes with polymorphism	58	48	106

In predicted probe	23	12	35

Outside the probe sequence	6	12	18

Both in and outside probe	29	24	53

No. of unigenes sequenced with no polymorphism anywhere	42	16	58

Approximately half (195/359) of the primers did not amplify products as expected. Primers from 27 genes (8%) could amplify a DNA fragment only from varieties in one cluster but not the other; therefore, sequence comparison between the two clusters was not possible. These markers can be run in an agarose gel and scored as dominant markers. The remaining 168 primers (46%) generated either multiple bands or did not amplify any product in varieties from both clusters.

### SNP Analysis and Mapping Using SNaPshot

SNPs were verified by the single-base extension method using the SNaPshot kit. Of the 58 genes found to contain SNPs between Ning 7840 and Clark, one SNP per gene was selected based on the DNA sequence surrounding it and its distance from other SNPs. Thirty-three SNP primers amplified SNPs that matched the sequence data (Table 2). Thirteen SNP primers gave either multiple peaks or no peak in at least one variety.

**Table 2:** Single nucleotide polymorphisms among six wheat genotypes using SNaPshot analysis.

	Ning 7840 and Clark Variety Pair	Other Variety Pairs	Total
No. of unigenes confirmed by SNaPshot	33	30	63

No. of unigenes with no suitable SNP primer	7	2	9

No. of SNP markers with multiple peaks in at least 1 variety	6	6	12

No. of failed SNP reactions (no peaks in at least 1 variety)	4	4	8

No data/design other primers	8	6	14

Forty-two SNP markers were further analyzed in 96 F_8–12 _recombinant inbred lines from the cross of Ning 7840/Clark; 28 were genotyped using SNaPshot, the rest as dominant markers in an agarose gel. Thirty-four markers were successfully integrated into the existing SSR map of the population (see additional file 1).

### A Map of Wheat SFPs

**Figure 2:**
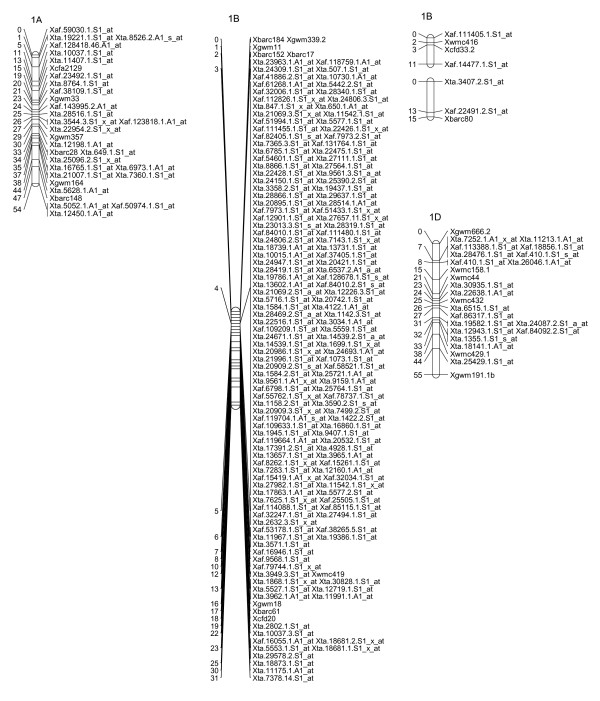
**A high-density map of wheat chromosome 1 consisting of 222 SFP and SSR markers in Ning 7840/Clark population**. Numbers to the left of each chromosome are interval distances in centimorgans. On the right of each chromosome are mapped markers; markers derived from the Affymetrix 'Ta.' probe sets were abbreviated as 'Xta' and those from 'TaAffx' as 'Xaf'.

**Figure 3:**
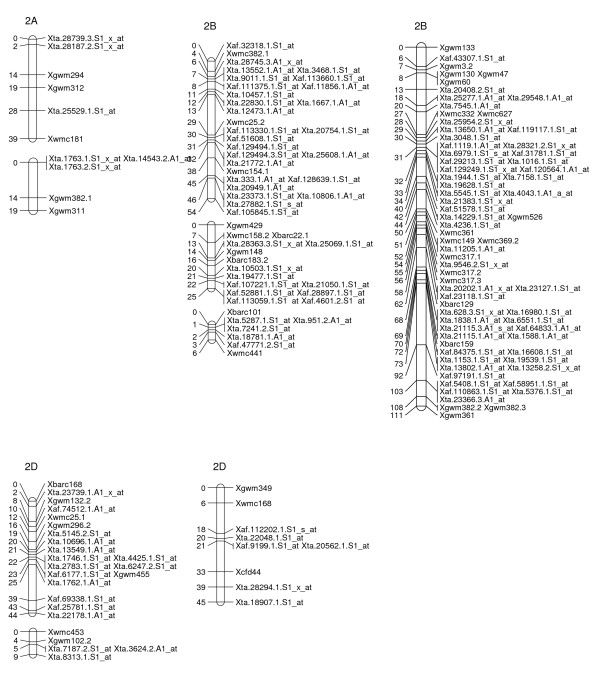
**A high-density map of wheat chromosome 2 consisting of 164 SFP and SSR markers in Ning 7840/Clark population**. Numbers to the left of each chromosome are interval distances in centimorgans. On the right of each chromosome are mapped markers; markers derived from the Affymetrix 'Ta.' probe sets were abbreviated as 'Xta' and those from 'TaAffx' as 'Xaf'.

**Figure 4:**
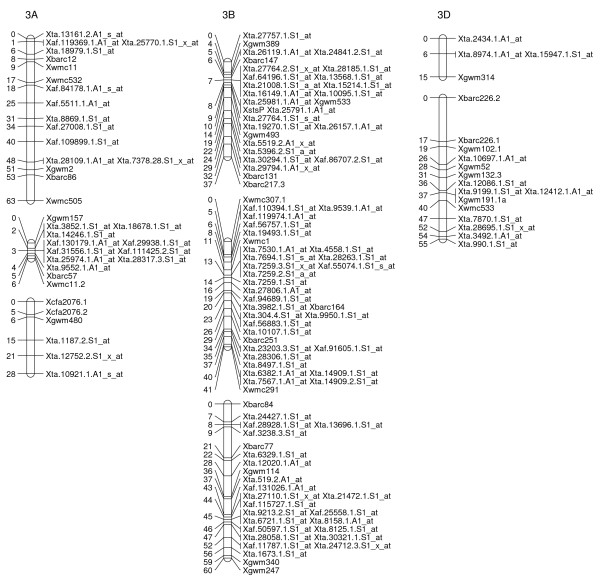
**A high-density map of wheat chromosome 3 consisting of 142 SFP and SSR markers in Ning 7840/Clark population**. Numbers to the left of each chromosome are interval distances in centimorgans. On the right of each chromosome are mapped markers; markers derived from the Affymetrix 'Ta.' probe sets were abbreviated as 'Xta' and those from 'TaAffx' as 'Xaf'.

**Figure 5:**
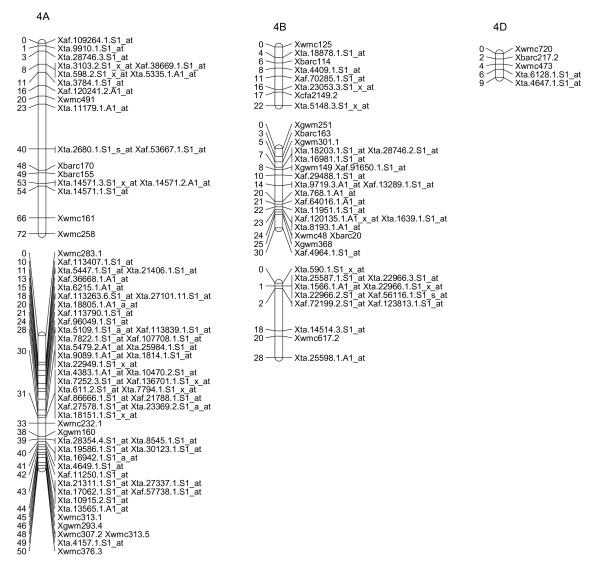
**A high-density map of wheat chromosome 4 consisting of 118 SFP and SSR markers in Ning 7840/Clark population**. Numbers to the left of each chromosome are interval distances in centimorgans. On the right of each chromosome are mapped markers; markers derived from the Affymetrix 'Ta.' probe sets were abbreviated as 'Xta' and those from 'TaAffx' as 'Xaf'.

**Figure 6:**
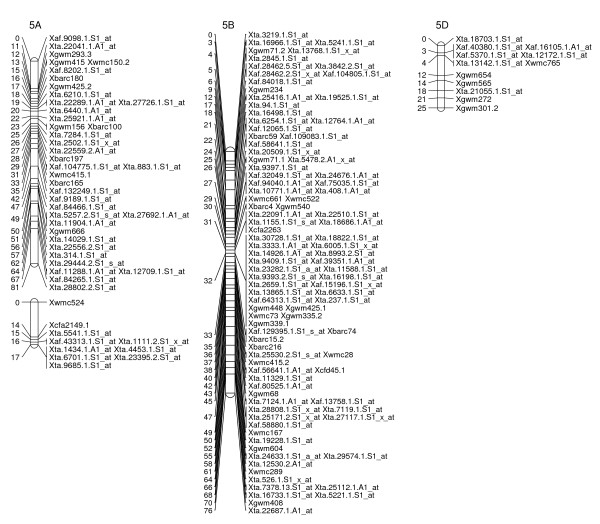
**A high-density map of wheat chromosome 5 consisting of 157 SFP and SSR markers in Ning 7840/Clark population**. Numbers to the left of each chromosome are interval distances in centimorgans. On the right of each chromosome are mapped markers; markers derived from the Affymetrix 'Ta.' probe sets were abbreviated as 'Xta' and those from 'TaAffx' as 'Xaf'.

**Figure 7:**
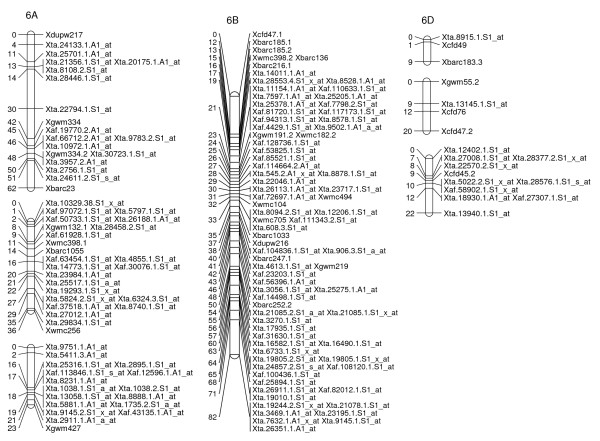
**A high-density map of wheat chromosome 6 consisting of 154 SFP and SSR markers in Ning 7840/Clark population**. Numbers to the left of each chromosome are interval distances in centimorgans. On the right of each chromosome are mapped markers; markers derived from the Affymetrix 'Ta.' probe sets were abbreviated as 'Xta' and those from 'TaAffx' as 'Xaf'.

**Figure 8:**
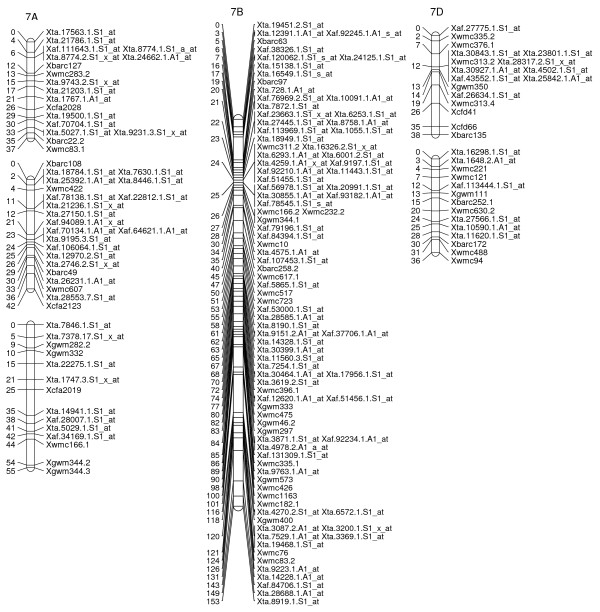
**A high-density map of wheat chromosome 7 consisting of 177 SFP and SSR markers in Ning 7840/Clark population**. Numbers to the left of each chromosome are interval distances in centimorgans. On the right of each chromosome are mapped markers; markers derived from the Affymetrix 'Ta.' probe sets were abbreviated as 'Xta' and those from 'TaAffx' as 'Xaf'.

**Figure 9:**
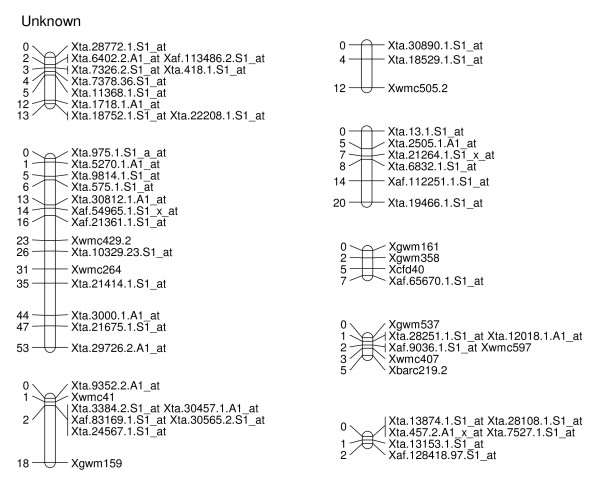
**Unassigned linkage groups consisting of 58 SFP and SSR markers in Ning 7840/Clark population**. Numbers to the left of each chromosome are interval distances in centimorgans. On the right of each chromosome are mapped markers; markers derived from the Affymetrix 'Ta.' probe sets were abbreviated as 'Xta' and those from 'TaAffx' as 'Xaf'.

To explore the possibility of directly mapping the SFPs, 71 RILs were analyzed on the Affymetix Wheat Genome Arrays. A total of 2,426 probe sets showed different intensity patterns between the parents, Ning 7840 and Clark, as evidenced by significant probe × variety interaction (*p *< 1e^-7^). Out of the 2,426 SFPs, 955 SFPs with at least 60 RIL calls were identified and 142 of these matched SFPs found in the first experiment. Nine hundred and twenty-three (97%) SFPs and 269 SSRs were mapped in 54 linkage groups and covered 1,944 cM genetic distance. Of the 53 linkage groups, 45 can be assigned to 21 chromosomes (Figures 2, 3, 4, 5, 6, 7, 8 and 9) according to previously reported SSR map information [25]. A total of 877 SFPs could be assigned to a chromosome location. About 63% of the SFP were mapped on the B genome, and only 10% were mapped in the D genome (Table 3). Chromosome 1B had the most SFPs, and 4D had the fewest. Chromosome arm 1BS had about 125 SFP markers that only spanned about 3 cM (Figure 2).

**Table 3:** Number of mapped markers per chromosome.

Chromosome	Genome A		Genome B		Genome D		Total	
	
	SFP^a^	SSR	SFP	SSR	SFP	SSR	SFP	SSR
1	25	6	154^b^	12^b^	19	6	198	24

2	6	4	92	29	23	10	121	43

3	24	12	70	18	11	7	105	37

4	59	13	30	11	2	3	91	27

5	35	13	74	23	7	5	116	41

6	52	8	61	16	12	5	125	29

7	38	16	68	24	15	16	121	56

Unknown							46	12

Total	239	72	549	133	89	52	923	269

^a ^SFP denotes an Affymetrix GeneChip probe set with at least one polymorphic probe.

^b ^Genomic *in situ *hybridization indicated that Ning 7840 carries a translocation where the short arm of wheat chromosome 1B is replaced by the short arm of rye chromosome 1R.

Of the 877 mapped SFPs, 92 corresponded to ESTs that have been previously bin-mapped to specific chromosome locations [26]. Comparison of map locations of the mapped SFPs from this study with those ESTs from the previous reports indicated that 8 SFPs (9%) from our study were not assigned to a known chromosome due to lack of SSR anchor markers in the group. Among the remaining 84 EST-mapped SFPs, 59 (70%) were assigned to the same chromosome positions as that reported previously; only 25 (30%) of the SFPs were mapped to either a homoeolog in another genome (11/25) or to a non-homoeologous chromosome (14/25). Therefore, map positions of most of SFPs derived from Affymetrix Wheat Genome Arrays were in agreement with the EST physical mapping results.

Of the 34 SFP-derived SNP markers that were mapped on 96 RILs in the first experiment, 28 (82%) were detected as SFPs on 71 RILs in the second experiment. Of the six SFPs that were not found in the second experiment, four showed significant probe × variety interactions, but were rejected because they did not meet the threshold of 60 good allele calls. Marker *Xta.5479.2.A1_at *in the second map was found near the location of the related marker *Xta.5479.1.A1_x_at *in the first map and this suggested that the SFP was reproducibly detected, albeit by a related probe set.

Individual RIL genotype calls using SFPs were compared to RIL genotype calls obtained previously by the SNaPshot method. Out of 28 SFPs that could be compared, three SFPs had allele calls that were identical with SNP analysis using SNaPshot, whereas 20 SFPs had 1% to 5% calls different from the SNaPshot results. Four markers had 8% to 13% calls that did not match, and one SFP (*Xta.25921.1.A1_at*) had 20% mismatch calls. Except for *Xta.25921.1.A1_at*, all SNP markers were mapped within 6 cM of the corresponding SFP marker positions.

## Discussion

This study demonstrated that the Affymetrix GeneChip Wheat Genome Array can be successfully used to identify and map SFPs in the large allohexaploid genome of wheat. A total of 1209 unique SFPs were identified among two bread wheat varieties in two experiments. An estimated 54% of the wheat SFPs was associated with sequence polymorphisms in the probe sets. This is only slightly lower than the 60% reported in barley [17] and indicates that SFPs are a valuable source of SNPs and other sequence polymorphisms in wheat. A high resolution map was produced containing 877 SFPs that were located on each of the 21 wheat chromosomes using SSR framework markers. We believe this is the first report of an SFP-based genetic map that covers all three wheat genomes. This map will be a useful tool for SNP discovery, QTL mapping, marker-assisted breeding, and functional analysis of wheat genes. In the course of this study, a total of 492 sequence polymorphisms were also identified in bread wheat germplasm.

### SFP Discovery

Two approaches to identify high quality SFPs were used. In the first method, cRNA from a panel of six diverse bread wheat varieties were hybridize to microarrays to identify unigene probe sets that had a significant (*p *< 1e^-10^) probe × variety interaction in the ANOVA. Cluster analysis was used to select only those probe sets that formed two robust clusters of alleles, with each cluster containing at least two varieties. This conservative algorithm was expected to produce robust, biallelic SFPs that are widespread in bread wheat germplasm; 396 SFPs were identified by this method.

In the second method, cRNA from parents of the cross Ning 7840/Clark was hybridized to microarrays to identify probe sets that had a significant (*p *< 1e^-7^) probe × variety interaction. Although only two varieties were used, the lower *p*-value criterion allowed the identification of a large number of SFPs. For each probe set, alleles in the 71 RILs were matched with either parental alleles and only those probe sets with at least 60 good allele calls were retained. This conservative algorithm was expected to produce robust Mendelian SFP marker loci. We identified 955 SFPs using this method. The requirement for good allele calling appeared to be a powerful filter for rejecting low quality SFPs because 97% (923/955) of SFPs were successfully assigned to a linkage group and 92% (877/955) were anchored to a chromosome location (Table 3, Figures 2, 3, 4, 5, 6, 7 and 8).

SFPs from both of the methods were mapped on the Ning 7840/Clark RILs, and this provided an opportunity to compare the results. Of the 34 SFP-derived SNPs mapped with the first method, 28 (82%) were also detected in the second method. Furthermore, they mapped to the same chromosome locations with only slight variations. This high concordance suggests that both methods were very successful in identifying reproducible SFPs. The first method appeared to be more conservative and more likely to identify broadly useful polymorphisms. However, the second method might be more cost effective in terms of cost per data point because it produced a large number of markers that were ready to be mapped in a linkage map. The Affymetrix Wheat Genome Array has numerous unigene probe sets with individual probes that perfectly match and hybridize to transcript sequences from more than one unigene. This is potentially problematic for hexaploid wheat where up to three homoeologs may have closely related sequences that could hybridize to the same 25-mer probe feature on the microarray. Matching sequences in paralogous gene copies could also cause hybridization to the same feature. This nonspecificity could reduce the signal-to-background ratio of the SFP and make it difficult to distinguish the difference in signal intensity between the polymorphic alleles. A low signal-to-background ratio would tend to reduce statistical significance of the probe × variety interaction. Nonspecificity of probes might also lead to misidentification of homoeologous sequence variants as multiple alleles of the locus. Both methods in this study were designed to reject SFPs with low statistical significance or multiple alleles.

There were at least 15 instances of cosegregation of SFPs from related unigenes (Figures 2, 3, 4, 5, 6, 7, 8 and 9). Some of these cases of cosegregation could be due to closely linked genes or members of a gene family that were not resolved into separate loci due to the limited size of the mapping population. Others could be due to transcripts that hybridize to more than one probe set. For example, marker *Xta.1763.1.S1_x_at *cosegregates with *Xta.1763.2.S1_x_at *on chromosome 2A (Figure 3). The probe sets associated with the two markers each have two probes that overlap and share some sequence with the other unigene probe set. Therefore, the same SFP could be detected by both unigene probe sets if it occurs in the shared overlapping probe sequences. A third cause of cosegregation could be polymorphisms that affect more than one probe in the unigene probe set. For example, related SFP markers *Xta.7259.1.S1_at*, *Xta.7259.2.S1_a_at*, *Xta.7259.3.S1_x_at *and *Xaf.55074.1.S1_s_at *all mapped within 1 cM of each other on chromosome 3B (Figure 4). Although each has sequences that match unigene Ta.7259.1, there is no single probe sequence that is common to all four probe sets. Therefore, a polymorphism in a single probe feature could not account for the observed pattern. However, a large indel or alternative splicing could result in a polymorphism that spans multiple features and be detected by all four probe sets. In all of these cases, the SFP should be mapped on the correct chromosome location because the RILs would be scored correctly for the underlying polymorphic locus. However, some of the unigene markers may appear to be in the wrong map location because their probe sets are actually detecting a polymorphism in a different member of the gene family. If related marker cosegregation is due to multiple probes detecting the same polymorphic locus, the number of unique SFPs would need to be adjusted downward.

Among the 164 unigenes with SFPs that were sequenced in this study, 46% did not have sequence polymorphisms inside the probes (Table 1). This cannot be attributed solely to SFP discovery errors because 82% of SFPs mapped in the first experiment were verified in the second experiment. Sequencing results in this study are similar to those of Rostoks et al. who reported that 40% of SFPs between barley varieties Morex and Golden Promise did not reveal any sequence polymorphisms in the probes [17]. Luo et al. reported that 64% of the SFPs identified between barley varieties Morex and Steptoe did not contain sequence polymorphisms in any of the features [12]. The majority of the SFPs were thought due to *cis*-acting expression regulators. One possible explanation for the monomorphic SFP sequences is alternative splicing, which may occur in more than one-fifth of plant genes [27]. Only PCR primers that produced a single band were used in this study, but it is possible that the wrong comigrating homolog was amplified and sequenced in some cases. The SFP discovery methods used in this study were designed to ignore differences in gene expression levels, known as GEMs. However, the analyses assumed that the log intensities of individual probes in a probe set are all a linear function of transcript abundance. When this is not the case for any reason, differences in expression level can result in SFPs.

### SFP Mapping

The new SFP/SSR map in this study was constructed from an existing Ning 7840/Clark mapping population that is segregating for many important agronomic traits such as yield, bread quality, growth habit, and disease resistance [28]. A relatively small population of 71 RILs was used for scoring SFPs because of the high cost associated with Affymetrix array analysis. Therefore, the map resolution for SFPs is not as high as desired. However, the first high-density genetic map of wheat SSRs was constructed with only 70 RILs [29,30]. In this study, 269 SSR framework markers were scored on 96 RILs and linkage relationships were verified against a high density consensus SSR map [25]. The SSR anchor markers allowed the confident assignment of all but a few small linkage groups to chromosomes.

By comparing map positions of a select set of markers in this study with the physical position of previously mapped corresponding ESTs [26], the chromosome positions of 70% of SFPs in this study were found to be in agreement with those in previous reports. About 13% of SFPs were mapped to homoeologous positions, and only 17% were mapped in nonhomoeolog positions. The discrepancies could be due to *trans*-acting regulatory elements, cross hybridization among homoeologs, incorrect map positions of the SFPs, or incorrect bin-assignment of the ESTs in previous reports. Given the many possible biological and technical sources of discrepancies, 70% concordance suggests that the SFP mapping was accurate in this study.

The distribution of SFPs among the three genomes was unequal (Table 3). The B genome had the most SFPs, and the D genome had the least, which is in agreement with a previously reported SSR map [29], although that map was constructed using a mapping population derived from a synthetic hexaploid wheat as one parent. The low level of polymorphism in the D genome is due to genetic bottlenecks encountered during the evolution of bread wheat [9,31]. Targeting SFPs in synthetic hexaploid wheats could improve the SFP map resolution of the D genome.

The mapping cross parent Ning 7840 carries the 1RS.1BL centric translocation where wheat chromosome arm 1BS is replaced by rye chromosome arm 1RS. Although 1BS and 1RS are syntenic, sequence polymorphism is high and recombination between 1RS and 1BS does not occur. Using 1BS translocation and aneuploid stocks, Bhat et al. used the wheat GeneChip microarray to identify probe sets that showed between-probe variance changes related to the presence of 1BS [32]. This novel approach demonstrated that SFPs associated with 1RS.1BL are detectable with the wheat GeneChip. In our study, chromosome 1B had 154 SFPs, which was the highest for any chromosome (Table 3). The majority of these SFPs cosegregated or were tightly linked. Unequal number of good allele calls probably explained why cosegregation was not perfect in the nonrecombining segments. Chromosome arm 1BL, which does recombine, accounted for additional map distances between markers on 1B. The results for the 1RS.1BL translocation were consistent with expectations and verified the power of these methods to detect and map SFPs in alien chromosome segments. However, recombination suppression that is often associated with alien segments limited the map resolution in that region.

### SNP Discovery

In wheat, SNP frequency estimates from different studies range from one per 335 bp [33] to one every 540 bp [10]. A SNP frequency of one in 112 bp was detected in this study, using genes that were prescreened for SFPs using the Affymetrix Wheat Genome Arrays. Although these studies used different varieties and methods, it appears that SFP screening significantly enriched SNPs.

Using conservative algorithms, a total of 1209 unique SFPs were identified between two bread wheat varieties in this study. More SFPs will be needed for large scale SNP discovery. Less conservative and more sophisticated SFP detection algorithms could improve discovery rates by an order of magnitude. Increasing population size and hybridization quality may add more SFPs because more lines will give clear allele calls for more SFPs. In addition, more SFPs could be identified by using more varieties, tissues, developmental stages, or treatments to create more diverse mRNA pools that represented more genes.

The advantages of using SFPs for SNP discovery in wheat are: 1) high throughput gene mining, 2) no prior sequence information required for varieties to be tested, 3) SNPs in coding sequences may be relevant to trait phenotypes, 4) potentially low cost per SNP for direct mapping using Affymetrix GeneChip and 5) commercial service available for Affymetrix analysis and data are comparable among labs. Furthermore, scoring SFPs on mapping populations allows filtering for reproducible, Mendelian, genome-specific polymorphisms. The disadvantages are: 1) initial cost is high for microarrays, 2) RNA pools must be obtained from carefully controlled samples, 3) polymorphic intergenic regions and introns are not sampled, and 4) still need to sequence the selected genes to validate SNPs.

### SNP and SFP Genotyping

Single-base extension using the SNaPShot kit (Applied Biosystem, Foster City, CA) was the platform of choice for SNP genotyping in this study. It is ideal for small-scale SNP analysis because it requires only a minimum setup fee per marker and provides clear differentiation of two SNP alleles. A variety of high throughput SNP genotyping platforms have been developed for human medical applications and successfully applied in several plant species that have relatively simple genomes. However, complex hexaploid wheat genomes makes high throughput SNP analysis more difficult and therefore more research is needed to identify the best platform for high throughput SNP analysis.

In this study, 877 SFPs were used directly as markers in the Ning 7840/Clark mapping population. The cost of arrays, associated reagents, and labor was less than $1.00 per mapped data point, which is much less than that for SSR and RFLP markers. Therefore, it is a cost effective method for spontaneous discovery and mapping of SFP in a mapping population. In the mapping population, allele calls were reliable because clear allele calls were derived from 60 RILs and segregation was normal. Increasing the population size may further improve data quality. The use of SFPs instead of SNPs in genome-wide association studies and linkage disequilibrium mapping has been investigated in Arabidopsis [34]. However, caution will be needed if using wheat SFPs directly as markers in applications where unknown genetic factors could affect probe cross hybridization.

The Affymetrix Wheat Genome Array was designed for gene expression studies rather than genotyping. Accordingly, there is not a one-to-one relationship between unigenes and probe sets. The majority of probe sets contain overlapping probes. In addition, there are many related unigene probe sets, often homoeologs that share identical probes or partial probe sequences. A custom-designed wheat genotyping chip with less confounding of sequences could solve some of the SFP interference problems associated with direct use of Wheat Affymetrix GeneChip for SFP analysis.

## Conclusion

This study demonstrated that the Affymetrix GeneChip Wheat Genome Array can be successfully used to identify and map SFPs in the large allohexaploid genome of wheat. The high density map generated in this study will be a useful tool for SNP discovery, QTL mapping, marker-assisted breeding, and functional analysis of wheat genes.

## Methods

### Plant Materials

Six wheat varieties of diverse classes and origins and one mapping population were used in this study (Table 4). Chinese Spring is a popular wheat genetic material from which various aneuploid and deletion genetic stocks have been developed; it is widely used in wheat cytogenetics and genomics studies. Encruzilhada originated from South America and is moderately resistant to Fusarium head blight (FHB). Jagger is a hard winter wheat variety released from Kansas and has been widely used as a major parent in hard winter wheat breeding programs in the Great Plains. Opata 85 is a hard red spring wheat variety developed at the International Maize and Wheat Improvement Center (CIMMYT) that has slow rusting resistance and is a parental line of an International *Triticeae *Mapping Initiative (ITMI) mapping population. Ning 7840 is a hard red facultative Chinese variety with a high level of resistance to FHB and leaf and stripe rusts. Genomic *in situ *hybridization indicated that Ning 7840 carries a translocation where the short arm of wheat chromosome 1B is replaced by the short arm of rye chromosome 1R (Bernd Friebe and Li-li Qi, personal communication). Clark is a soft winter wheat variety released from Purdue University, Indiana, USA, with high yield potential, but it is susceptible to FHB and rusts. A mapping population of 96 RILs derived from the cross Ning 7840/Clark was used to construct an SSR linkage map and to map SNPs scored using SNaPshot analysis. A subset of 71 RILs randomly selected from the population was used for mapping SFP derived from Affymetrix analysis.

**Table 4:** Origin and pedigree of wheat varieties used in this study.

Variety	Country	Pedigree
Chinese Spring	China	Chinese landrace

Clark	USA	Beau//(65256A1-8-1/67137B5-16/4/Sullivan/3/Beau//5517B8-5-3-3/Logan)

Encruzilhada	Brazil	Fortaleza/Kenya Farmer

Jagger	USA	KS-82-W-418/Stephens

Ning 7840	China	Aurora/Anhui//Sumai 3

Opata 85	Mexico	Bluejay/Jupateco 73

For RNA isolation, 10 seeds per variety were planted in a 12.7 cm. × 12.7 cm. Dura-pot (Hummert Int., St. Louis, MO) containing Metro Mix 360 soil mix (Hummert Int., St. Louis, MO), and grown in a growth chamber at 20°C under 12 h of light and 15°C for 12 h under darkness. All varieties had two biological replicates (pots). Leaves and roots of five seedlings from each pot were collected when plants reached the three- to four-leaf stage. Collected roots were washed and blotted dry with paper towels. Tissues were immediately frozen in liquid nitrogen and stored at -80°C until use.

### Microarray Analysis

Probe sets from 55,052 transcripts spanning all 21 chromosomes of wheat are spotted on the Affymetrix GeneChip Wheat Genome Array. Each probe set has eleven 25-mer perfect match (PM) probes and eleven mismatch (MM) probes, which differ in only one base as internal controls. In this study, data from the MM probes were not used. Although a majority of the oligos spotted on the array are from *T. aestivum*, probe sets from *T. monococcum*, *T. turgidum *and *Ae. tauschii *are also included. Probe sets with the same unigene number are from related genes, and different members of the group are denoted after the decimal. Different alphabetic suffixes denote whether probe sets contain probes that are perfect matches to more than one exemplar. Detailed microarray data and protocols can be accessed at: 

Leaf and root tissues from bulks of five plants were pooled prior to RNA extraction. Total RNA was extracted from powdered tissues using Trizol reagent (Invitrogen, Carlsbad, CA), and the extracted RNA was further purified using RNeasy Midi Kit (Qiagen, Valencia, CA). RNA purity and integrity was checked using Agilent BioAnalyzer 2100 (Agilent Technologies, Santa Clara, CA). Microarray analysis was done at the Big Red Spots Microarray Facility, Cornell University, Ithaca, New York, USA, and Weill Medical College, New York, New York, USA, following standard methods for cRNA synthesis, labeling, hybridization, and data acquisition as described in the Affymetrix manual (Affymetrix, Foster City, CA). For each variety, there were two biological (pools of five plants each) and two technical replications. For each RIL, only a single rep was processed.

### SFP Prediction between Wheat Varieties

The *.CEL files and *.RPT files generated by Affymetrix software from scans of the arrays were used as input. The RPT files contained several summary and data quality parameters for each array, such as percentage present calls, 3'/5' ratios of housekeeping genes, background signal, and scale factor. These parameters were consistent and within expected ranges for all arrays. The CEL files contained the individual probe signal intensities, which were imported into SAS datasets for further analysis. The entire study used a total of 22 Affymetrix Wheat Genome Arrays with four replications each of five varieties and two technical replications of a sixth (Clark). We planned to use two arrays from an earlier run representing a second biological replication of Clark but determined these to be of lower quality and thus excluded them from the study.

The method described by Kirst et al. using a test of probe × variety interaction as an indication of the presence of a SFP was used to analyze the PM probe signal intensities [35]. First, the log base 2 of the PM signal intensity (log_2_pm) was calculated and standardized to a mean of zero for each probe set by array combination. Then for each probe set individually, SAS PROC MIXED [24] was used to fit a linear model with probe, variety, and probe × variety as fixed effects and array nested in variety as a random effect. The mean log_2_pm for each probe × variety combination was output using the lsmeans option. Any probe set with a probability of less than 1e^-10 ^for the F test of the probe × variety effect was selected as potentially polymorphic. Next, probe sets that had fewer than three probes with raw signal intensity over 200 on any array were deleted from the list of candidates. This step avoided spurious interactions that could have resulted from lack of expression in one or more lines.

For each remaining probe set, varieties were grouped into two clusters using SAS PROC FASTCLUS [24]. This procedure forms clusters using the Euclidean distance between probe sets based on the lsmean probe estimates from the previous step. The ratio of between cluster variance to within cluster variance was used to help determine which probe sets gave the clearest indication of a SNP. By examining graphs of probe means vs. probe position in the probe set for a sample of probe sets, an Rsq ratio of four or greater was determined to give a clear indication of different patterns of hybridization that would be likely to be caused by a SNP. The candidate list was further narrowed by including only those probe sets with a minimum of two varieties in the smaller of two clusters to find SNP with intermediate allele frequencies.

### SFP Analysis in RILs

Genotypes were assigned to 71 RILs developed from Ning 7840/Clark using expression microarray data in the following manner. In the first step of the process, probe sets were identified that showed a different hybridization pattern for each of the two parents. In the second step, for each RIL in turn, the parent most closely matching the RIL pattern was identified. If the RIL could not be matched to one parent with a predetermined level of confidence, that data point was set to missing.

To evaluate the parents, all available microarrays determined to be of good quality were used. For Ning 7840, that was comprised of four microarrays evaluated at the Big Red Spots facility and two evaluated at Weill Medical College or two technical reps each of three biological reps. For Clark, two technical reps of a single biological rep were processed at Big Red Spots, and two technical reps of another biological rep were processed at Weill. A single rep of each RIL was processed at Weill. The method described by Kirst et al. was used to identify probes that could distinguish Ning 7840 from Clark [35]. To eliminate comparisons that differed only by RNA expression level, the model, intensity = probe + variety + array:variety + probe × variety was fit to each probe set, and an F-test of the null hypothesis H_0_: probe × variety interaction effect = 0 was performed. A total of 2,426 probe sets for which the null hypothesis was rejected at a *p*-value of 1e^-7 ^were identified. That *p*-value corresponds roughly to a Bonferroni-corrected alpha level of 0.01.

For those probe sets, intensities for all the microarrays of each parent were averaged to create a single average intensity for each probe and parent combination. For each RIL and probe set combination, the following linear model was fit to the RIL probe intensities and the Clark probe intensities: Intensity = Variety + Probe, where "Variety" can be either RIL or Clark and Probe belongs to the probe set being analyzed. The same model was fit separately for the RIL and Ning 7840. If the pattern of probe intensities for the RIL was most like Clark, the model would be best fit by the Clark data, and that model would have a smaller error than the Ning 7840 data fit to the same model. Because the error mean squares from the models are estimates of the error variance, the ratio of the mean squares forms an F-test for equality of the variances and the *p*-value calculated from it provides a measure of confidence that the RIL had been assigned to the correct parent. If (MS Error Clark)/(MS Error Ning 7840) > 1 and the *p*-value associated with the F-test was less than 0.1, the RIL was assigned a value of *A *(= Ning 7840 allele) at that probe set. If the ratio was less than one and the F-test of (MS Error Ning)/(MS Error Clark) had a *p*-value less than 0.1, the RIL was assigned a value of *B *(= Clark allele). Otherwise, the genotype was considered to be missing. The analysis was run using the R statistical program [36].

### DNA Sequencing

Genomic DNA was extracted from leaf tissues of all six varieties following the CTAB protocol of Saghai-Maroof et al. [37]. To confirm the presence of SNPs, variety pairs were selected for sequencing based on clustering results. Because RILs of Ning 7840/Clark were available, the two parents were chosen for SNP validation whenever they were assigned to different clusters; otherwise, other variety pairs were selected. PCR primers were designed to bind at least 100 bases upstream or downstream of the probes predicted to contain SFPs. Primer pairs with a predicted range of 300–500 bp were designed based on the wheat unigene consensus sequence (Affymetrix) using the computer program PerlPrimer V1.1.9 (Marshall 2004). The PCR mix contained 1× ammonium sulfate buffer (Bioline, Randolph, MA), 2 mM MgCl_2_, 0.2 mM of each dNTP, 0.5 uM of each primer, 100–200 ng DNA, and 1 unit Taq polymerase (Promega, Madison, WI) in a reaction volume of 40 μl. Touchdown PCR was carried out in a PTC-100™ Thermal Cycler (Bio-Rad, Hercules, CA) programmed as follows: 5 min at 94°C, 10 cycles of 20 s at 94°C, 20 s at 59–65°C (temperature at 5° higher than the annealing temperature in the 35-cycle stage) minus 0.5°C per cycle, 2 min at 72°C, followed by 35 cycles of 20 s at 94°C, 20 s at 54–60°C depending on the primer's annealing temperature, and 2 min at 72°C with a final extension of 10 min at 72°C. PCR products were visualized in a 2% agarose gel, and singleton bands were selected for sequencing. Reactions that generated multiple or no bands were reamplified using higher or lower annealing temperatures, respectively. Amplicons purified by GenCatch PCR Clean-up Kit (Epoch Biolabs, Houston, TX) were sequenced on an ABI 3730 DNA Analyzer using BigDye Terminator V1.1 (Applied Biosystems, Foster City, CA) at the Kansas State University Sequencing and Genotyping Facility (Manhattan, KS, USA). DNA sequence data was viewed using the DNA Sequencing Analysis Software V5.2 (Applied Biosystems, Foster City, CA) and checked manually for SNPs.

### SNP Confirmation Using SNaPshot

SNP genotyping was done following the SNP primer design guidelines and SNaPshot protocol of the manufacturer (Applied Biosystems, Foster City, CA) with few modifications. SNP primers were 18–24 bases long with the 3' end of the primer directly flanking the SNP. All primers had a *Tm *of at least 50°C, and primers were checked for possible extendable primer-dimer formation using PerlPrimer V1.1.9 [38]. The template used for SNP primer extension (second PCR) was the amplicons used for sequencing. Second PCR was done in a 10-μl reaction with 1 μl reaction mix, 0.2 μM primer, and 1 μl PCR product from first PCR as template. Samples were subjected to 25 cycles of 96°C for 10 s, 50°C to 60°C for 5 s (depending on primer annealing temperature), and 60°C for 30 s in a PTC-100™ Thermal Cycler (Bio-Rad, Hercules, CA). Following the incubation of PCR products with 1 unit of shrimp alkaline phosphatase (USB Corporation, Cleveland, OH) to remove unincorporated ddNTPs, 1 μl PCR product was analyzed on a 3730 DNA Analyzer (Applied Biosystems, Foster City, CA) after it was mixed with Hi-Di formamide and GeneScan 120 LIZ size standard (Applied Biosystems, Foster City, CA). Data were scored using GeneMarker software (Soft Genetics, State College, PA). Allele calls were compared with sequencing results.

To map SNPs in a genetic map, DNA was isolated from 96 individuals of an F_8–12 _Ning 7840/Clark RILs according to Saghai-Maroof et al. [38]. The mapping population was genotyped using 42 markers. Among them, 28 SNP were genotyped by SNaPshot and 14 by agarose gel (dominant markers). For dominant markers, PCR products were resolved in a 2% ethidium bromide-stained agarose gel in 1× TAE buffer. For SNaPshot, the first PCR product was cleaned up by treating 5 μl of the amplicon with 1.66 unit SAP and 0.66 unit exonuclease I (USB Corporation, Cleveland, OH). The reaction was incubated at 37°C for 1 h and deactivated at 75°C for 15 min.

### Map Construction

A total of 280 polymorphic SSR markers were used to construct the SSR map. SSR PCR was performed in a volume of 25 μl in a MJ PTC-200 Thermal Cycler (Bio-Rad, Hercules, CA). The reaction mixture contained 250 nM of each primer, 0.2 mM of each dNTP, 1.5 mM MgCl_2_, 1 unit *Taq *polymerase, and 50–100 ng of template DNA. The PCR reaction was incubated at 94°C for 3 min, followed by 45 cycles of 1 min at 94°C, 1 min at 50, 55 or 60°C (depending on the annealing temperature of each SSR primer pair), and 2 min at 72°C with a final extension at 72°C for 10 min. Samples were run in an ABI 3730 DNA Analyzer with 500 LIZ as size standard (Applied Biosystems, Foster City, CA) and analyzed using GeneMarker software (Soft Genetics, State College, PA).

SSR data were scored as either *A *for Ning 7840 allele, *B *for Clark allele or *H *for heterozygote. To ensure accurate scoring, all markers were scored at least twice. Loci with ambiguous bands were scored as missing data. The SNP or SFP data were combined with SSR data and the linkage map was constructed using JoinMap 3.0 (Kyazma, Wageningen, The Netherlands) with Kosambi mapping function and LOD value of at least 3 and maximum recombination frequency of 0.4.

## Authors' contributions

ESB GHB RLB ANB and PJB conceived and designed the experiments. PJB analyzed the microarray data. HXM carried out the SSR marker analysis. ANB and SWH did the sequencing. ANB performed the RNA extractions, SNaPshot analysis and mapping. PJB and GHB contributed reagents/materials/analysis tools. ANB PJB GHB and RLB wrote the paper. All co-authors provided inputs to improve the manuscript.

## Supplementary Material

Additional file 1 **Partial linkage map consisting of SNP and SSR markers in a Ning 7840/Clark mapping population**. This file contains linkage groups of SNP and SSR markers in a Ning 7840/Clark mapping population. Numbers to the left of each chromosome are interval distances in centimorgans. On the right of each chromosome are mapped markers; SNP markers derived from the Affymetrix 'Ta.' probe sets were abbreviated as 'Xta' and those from 'TaAffx' as 'Xaf'. Click here for file
