# Supplementary material for: Discovery and mapping of single feature polymorphisms in wheat using Affymetrix arrays
Source: BMC Genomics. 2009 May 29;10:251. doi: 10.1186/1471-2164-10-251 (PMC2698007; doi:10.1186/1471-2164-10-251)
Supplement: Supplementary file 1 [file 1471-2164-10-251-S1.rtf]

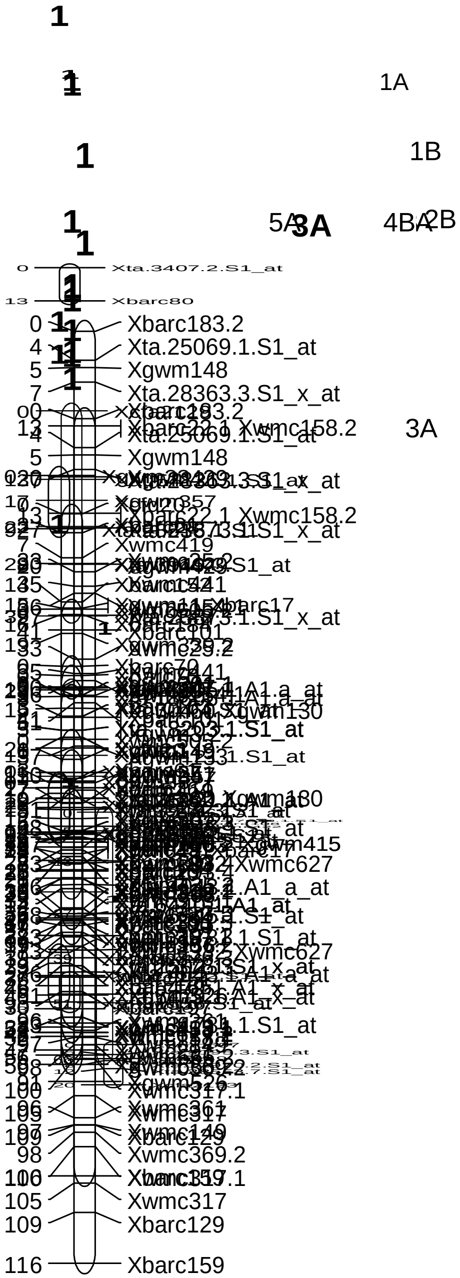
1A
1B
3A
0 Xta.3407.2.S1_at
13 Xbarc80
0 Xbarc183.2
4 Xta.25069.1.S1_at
5 Xgwm148
7 Xta.28363.3.S1_x_at
98 Xwmc369.2
105 Xwmc317
109 Xbarc129
116 Xbarc159

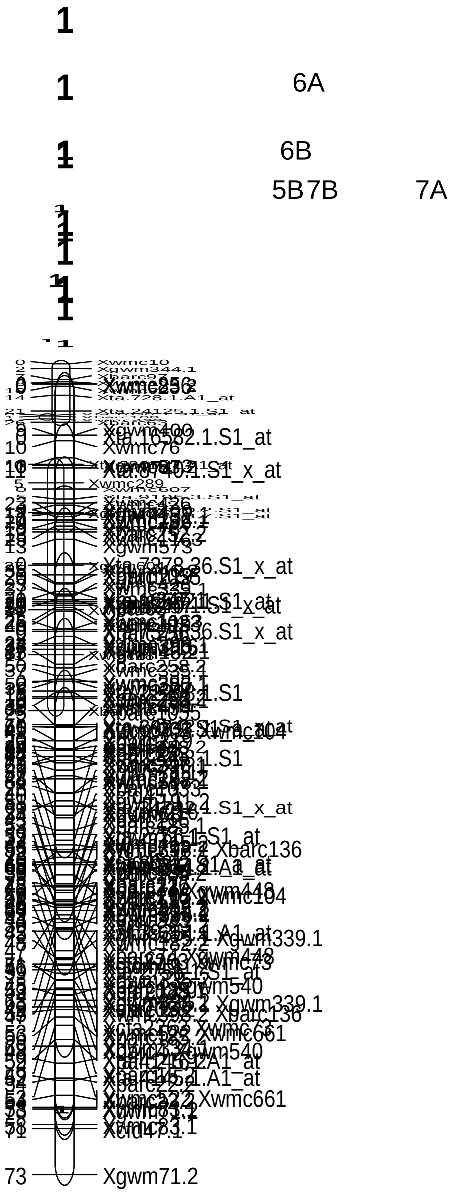
6A
6B
5B
7B
7A
73 Xgwm71.2

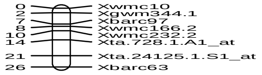
0 Xwmc10
2 Xgwm344.1
7 Xbarc97
8 Xwmc166.2
10 Xwmc232.2
14 Xta.728.1.A1_at
21 Xta.24125.1.S1_at
26 Xbarc63
